# Analysis of Volatile Compounds in Exhaled Breath Condensate in Patients with Severe Pulmonary Arterial Hypertension

**DOI:** 10.1371/journal.pone.0095331

**Published:** 2014-04-18

**Authors:** J. K. Mansoor, Edward S. Schelegle, Cristina E. Davis, William F. Walby, Weixiang Zhao, Alexander A. Aksenov, Alberto Pasamontes, Jennifer Figueroa, Roblee Allen

**Affiliations:** 1 Department of Physical Therapy, University of the Pacific, Stockton, California, United States of America; 2 Department of Anatomy, Physiology and Cell Biology, University of California Davis, Davis, California, United States of America; 3 Department of Mechanical and Aerospace Engineering, University of California Davis, Davis, California, United States of America; 4 Department of Medicine, University of California Davis Medical Center, Sacramento, California, United States of America; Keio University School of Medicine, Japan

## Abstract

**Background:**

An important challenge to pulmonary arterial hypertension (PAH) diagnosis and treatment is early detection of occult pulmonary vascular pathology. Symptoms are frequently confused with other disease entities that lead to inappropriate interventions and allow for progression to advanced states of disease. There is a significant need to develop new markers for early disease detection and management of PAH.

**Methodolgy and Findings:**

Exhaled breath condensate (EBC) samples were compared from 30 age-matched normal healthy individuals and 27 New York Heart Association functional class III and IV idiopathic pulmonary arterial hypertenion (IPAH) patients, a subgroup of PAH. Volatile organic compounds (VOC) in EBC samples were analyzed using gas chromatography/mass spectrometry (GC/MS). Individual peaks in GC profiles were identified in both groups and correlated with pulmonary hemodynamic and clinical endpoints in the IPAH group. Additionally, GC/MS data were analyzed using autoregression followed by partial least squares regression (AR/PLSR) analysis to discriminate between the IPAH and control groups. After correcting for medicaitons, there were 62 unique compounds in the control group, 32 unique compounds in the IPAH group, and 14 in-common compounds between groups. Peak-by-peak analysis of GC profiles of IPAH group EBC samples identified 6 compounds significantly correlated with pulmonary hemodynamic variables important in IPAH diagnosis. AR/PLSR analysis of GC/MS data resulted in a distinct and identifiable metabolic signature for IPAH patients.

**Conclusions:**

These findings indicate the utility of EBC VOC analysis to discriminate between severe IPAH and a healthy population; additionally, we identified potential novel biomarkers that correlated with IPAH pulmonary hemodynamic variables that may be important in screening for less severe forms IPAH.

## Introduction

An important challenge to pulmonary arterial hypertension (PAH) diagnosis and treatment is the early detection of occult pulmonary vascular pathology. Despite recognized risk factors for the disease, patients often present clinically only after a prolonged interval of symptoms. These symptoms are frequently confused with other disease entities, sometimes leading to inappropriate interventions and further progression to advanced states of disease. Even with proper diagnosis and therapy, the ability to judge the effectiveness of intervention is limited by the lack of assessment tools and biological markers.

Assessment tests such as the New York Heart Association (NYHA) Functional Classification and the 6 minute walk distance (6MWD) and biomarkers such as brain natriuretic peptide (BNP), though helpful, are limited in their ability to show PAH disease progression or quiescence. There is an unmet need to develop new markers for PAH disease detection and management that are valid, reliable and simple to obtain. Analysis of exhaled breath has been used to detect and monitor diverse pulmonary and systemic diseases such as oxidant-induced airway injury [1], aspirin-induced asthma [2], lung cancer [3], [4] COPD [5], tuberculosis [6], lung transplant rejection [7], breast cancer [8], heart transplant rejection [9], diabetes mellitus [10], and unstable angina [11].

Investigation in our laboratory using non-invasive exhaled breath condensate (EBC) analysis has shown that vascular endothelial growth factor, leukotriene B_4_, prostaglandin E_2_, isoprostane, nitrates and nitrites can be isolated from EBC in humans exposed to altitude [12] and ozone [1]. The purpose of this study was to analyze the metabolic signature of volatile organic compounds (VOC) in EBC in patients with class 3 and 4 idiopathic PAH (IPAH) using gas chromatography/mass spectrometry (GC/MS). We compared this metabolic signature against age-matched healthy controls. It is hoped that this analysis is a first step in identifying new markers that can potentially be used to screen for less severe IPAH (classes I and II).

## Methods And Materials

### Ethics Statement

This study was approved by the University of California, Davis, Office of Human Research Protection Institutional Review Board (protocol #200917227-1). Written informed consent was obtained from participants and the study was conducted according to principles expressed in the Declaration of Helsinki.

### Subjects

A total of 60 subjects ages 32–78 were recruited, with 30 healthy control subjects (8 males, 22 femlaes) and initially 30 functional class 3/4 IPAH who had undergone diagnostic right heart catheterization. The data from 3 IPAH subjects were insufficient for analysis reducing the final the IPAH group to 8 males and 19 females. Potential IPAH subjects were excluded if they were classified as functional class I/II IPAH or had anorexic drug-induced PAH, were HIV positive, were pregnant, had congenital heart disease, were currently smoking or had collagen-vascular disease. Control subjects were all non-smokers and pregnant women were excluded. An attempt was made to match age and body mass index (BMI).

### Research Design and Data Collection

Subjects had their exhaled nitric oxide (ExNO) measured followed by collection of EBC. ExNO was measured with a chemiluminescence nitric oxide analyzer (Sievers, Boulder, CO) using the restricted breath technique at flow rate of 50 ml/s [13]. Historical 6MWD, New York functional class, BNP, hemodynamic measures and pulmonary function data was extracted from IPAH patients' medical records for correlation analysis.

EBC samples were collected as subjects sat quietly with a nose clip on breathing into the Jaeger EcoScreen (Viasys Healthcare, Conshohocken, PA) apparatus [12]. 0.5 mL EBC samples were stored at −80°C in borosilicate vials and analyzed for VOC in a single batch using solid-phase microextraction (SPME). For SPME, EBC samples were thawed, 0.5 mL saturated NaCl solution added and sample vials placed on a chilled tray of a autosampler in randomized fashion. VOCs were sampled from the headspace using carboxen/polydimethylsiloxane df 75 µm (partially crosslinked [black hub]) SPME fibers (Supelco, St. Louis, MO) [14]. For analysis, EBC samples were transferred into a heater and agitated at 90°C with SPME tip exposed to the headspace. After sampling, the SPME was inserted into the heated inlet of a Varian 3800 GC with a 4000 Ion Trap MS (scanned mass-to-charge ratio (*m/z*) range 35–600 Th) equipped with an electron ionization source and a VF-5 ms 5% phenol/95% PDMS GC column (Varian, Walnut Creek, CA). The GC oven cycle was optimized for separation of benchmark human EBC samples.

### Data Analysis

#### Exhaled Breath Condensate Gas Chromatograms/Mass Spectra Analysis

In order to compare EBC GC/MS profiles of control and IPAH subjects, whole chromatogram analysis and specific peak comparisons were used to test for statistically significant differences (*p*<0.05) between groups. Baseline correction was applied to remove the shifted regions of all chromatograms. A peak was defined as a minimum height of 500 ion counts. GC column bleed peaks (siloxanes) or extraneous contaminats (e.g. phtalates) were excluded. The height values of all peaks in the chromatogram were summed for each individual chromatogram (Σ*h_i_*) and the average was calculated (*h*). A correction coefficient *r* was then calculated as r*_i_* = Σ*h_i_/h*. Each chromatogram was corrected by dividing the peak height value by r*_i_* in order to detect possible outliers and/or minimize the error in the sampling step. Then, peaks were normalized based on the height of the same peak of each sample to make the comparison on the same scale. Peaks with unacceptably low signal-to-noise ratios were not used. Across all of the chromatograms, 2668 peaks were identified using this approach.

To be considered unique, chromatograms of 70% of the subjects in a given group had to contain a given peak, while chromatograms of subjects in the other group did not contain the same peak. Peaks were considered in-common if chromatograms of subjects in both groups contained a given peak. We identified 62 unique peaks from the control group, 48 unique peaks from the IPAH group and 19 in-common peaks. In order to control for peaks that may have been affected by medications and/or dietary supplements being taken by the IPAH subjects, medications/dietary supplements were tabulated (see Table S1); 112 unique medications/dietary supplements were identified. A MANOVA was run using each medication/dietary supplement that was taken by 4 or more IPAH subjects as a grouping factor; if there was a statistically significant drug effect for a given peak, peak height data for the subjects taking that drug were removed from the database. After this, 5 peaks no longer met our criteria for an in-common peak and were removed from the original 19 in-common peaks for a total of 14 in-common peaks, and 16 peaks no longer met our criteria for a unique IPAH peaks and were removed from the original 48 unique IPAH peaks for a total of 32 unique IPAH peaks. MANOVA was used to determine if any of the 14 remaining in-common peaks were significantly different between groups.

#### GC/MS Autoregression/Partial Least Squares Regression Analysis

GC/MS data were converted into total ion count versus time. Each GC profile was composed of approximately 15,000 time points which covered 255 minutes. Baseline correction was applied to remove humps/plateaus in some of the chromatograms [15]. Auto-regression (AR) analysis was then used to reduce the number of variables needed to describe chromatograms from the original time scan number to one hundred AR coefficients [16], [17]. Auto-regression analysis has the advantage of reducing the dimensionality of chromatography data while reducing the effects of possible signal misalignment within different profiles [16], [17]. To visually and quantitatively compare the chromatography data of control and IPAH subjects, partial least square regression (PLSR) using AR coefficients was employed. PLSR further reduced the dimensionality from one hundred AR coefficients to two latent variables (PLSR components). The two PLSR components were then plotted against each other to examine separation within the data. This analysis was performed using Matlab software (MathWorks, Inc.; Natick, MA). This model was validated using the leave-one-out validation process. The results of this validation was used to calculate the sensitivity, specificity and positive and negative likelihood ratios.

#### Chemical Identification

Chemical identities of the 15 peaks unique to the control group with the highest ion counts and all of the unique IPAH and in-common peaks were explored. Where necessary, the Automated Mass Spectral Deconvolution and Identification System GC/MS analysis software (National Institute of Standards and Technology {NIST} v.2.64) was used to remove background noise and deconvolve peaks for co-eluting compounds. The MS spectra were compared against the NIST 2005 and Wiley 2009 MS libraries of deconvolve peaks for co-eluting compounds using NIST Mass Spectral Search Software v.2.0. The highest probability matches were considered and putative chemical identity was determined empirically by examining representative MS data and *m/z* in the data set. If the search produced a match with a probability greater than 80%, that match was considered to be the unknown compound (high confidence match). In some cases, no match was found or multiple chemical matches with very similar mass spectral fragmentation patterns and close match probability values (e.g., for isomers) were found. These were considered to be low confidence matches.

#### Correlation Analysis

The IPAH criteria endpoints of mean pulmonary arterial pressure (mPAP), pulmonary arterial wedge pressure (PAWP) and pulmonary vascular resistance (PVR) were correlated with all in-common or unique normalized peak heights of the IPAH group using Pearson-product moment correlation in order to identify peaks for inclusion in stepwise linear regression. mPAP, PAWP and PVR were also correlated with BNP and ExNO. All statistical analysis was performed using SPSS software version 21 (IBM Corp, Armonk, NY).

## Results

### Subject Characteristics

Table 1 shows the characteristics of the control and IPAH groups. The IPAH group mean values for PVR, PAWP, D_LCO_, CI and 6MWD (Table 2) met or exceeded the minimal criteria for IPAH [18]. The groups were similar in the distribution of males and females. There was no difference in age and height between the two groups, however, the IPAH group was significantly heavier and had greater BMIs than the control group. Additionally, the IPAH group had significantly lower exhaled nitric oxide values.

**Table 1:** Subject characteristics.

	Control (n = 30)	PAH (n = 27)
Gender (n): Male	8	8
Female	22	19
Age (yrs)	52.5±6.8	51.6±11.0
Height (m)	1.68±0.08	1.67.3±0.08
Weight (kg)	80.0±20.7	96.2±29.4*
BMI (kg/m^2^)	28.4±6.7	34.3±9.8*
Exhaled Nitric Oxide	32.4±26.7	16.3±11.8*

Values are means ± standard deviation;

* significantly different from control *p*≤0.05; BMI =  body mass index.

**Table 2:** Hemodynamic and clinical endpoints for IPAH subjects.

Endpoints	Values	n
MAP (mm Hg)	91.7±10.9	27
SBP (mm Hg)	123.0±14.9	27
DBP (mm Hg)	76.3±11.0	27
mPAP (mm Hg)	49.4±11.0	26
PAP_SYS_ (mm Hg)	74.1±15.2	26
PAP_DIA_ (mm Hg)	35.2±10.3	26
PAWP (mmHg)	10.6±4.2	26
PVR (mmHg/L/min)	705.2±295.6	26
P_RA_ (mm Hg)	10.2±5.1	25
Cardiac Index (L/min/m^2^)	2.32±0.53	26
Brain Naturetic Peptide (pg/mL)	207.1±245.9	24
6 Minute Walk Distance (m)	342.6±112.3	23
D_LCO_ (ml/min/mmHg)	20.4±7.7	21

Values are means ± standard deviation; MAP = mean arterial pressure; SBP = systolic blood pressure; DBP = diastolic blood pressure; mPAP = mean pulmonary artery pressure; PAP_SYS_ =  systolic pulmonary artery pressure; PAP_DIA_ = diastolic pulmonary artery pressure; PAWP =  pulmonary arterial wedge pressure; PVR = pulmonary vascular resistance; D_LCO_ =  lung cabon monoxide diffusing capacity. Note the high pulmonary arterial pressure and pulmonary vascular resistance characteristic of pulmonary arterial hypertension.

### EBC GC/MS Analysis

**Figure 1:**
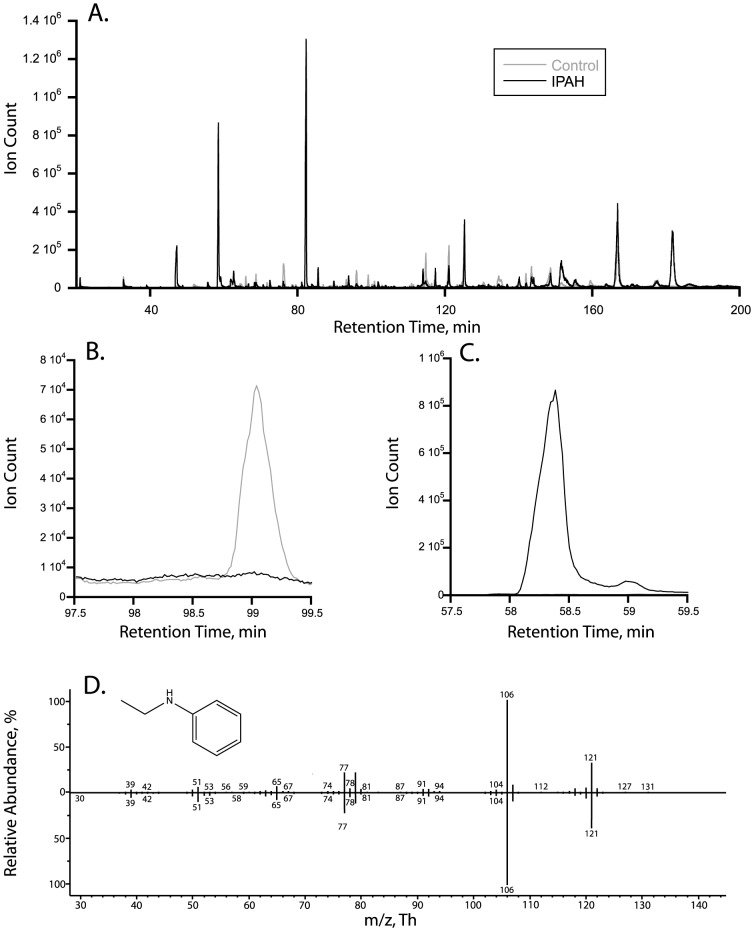
Representative gas chromatograms from a control subject (black line) and IPAH subject (grey line) (A) showing significantly different (*p*≤0.05) unique peaks for the control subject (B) and IPAH subject (C). An example of a head-to-tail comparison of an experimental mass spectrum (D) of one of the identified significantly different peaks unique for the IPAH group at a retention time of 81.436 min. (top) with a NIST/Wiley 2009 database search hit (bottom) identifying N-ethyl-Benzeneamine as giving the best match for the experimental spectrum.

Figure 1A shows a representative gas chromatogram for a control and an IPAH subject superimposed on one-another. Unique peaks found in the IPAH subject but not in the control subject and a peak found in the control subject but not in the IPAH subject are shown in figures 1B and 1C, respectively.

### GC/MS Autoregression/Partial Least Squares Regression Analysis

**Figure 2:**
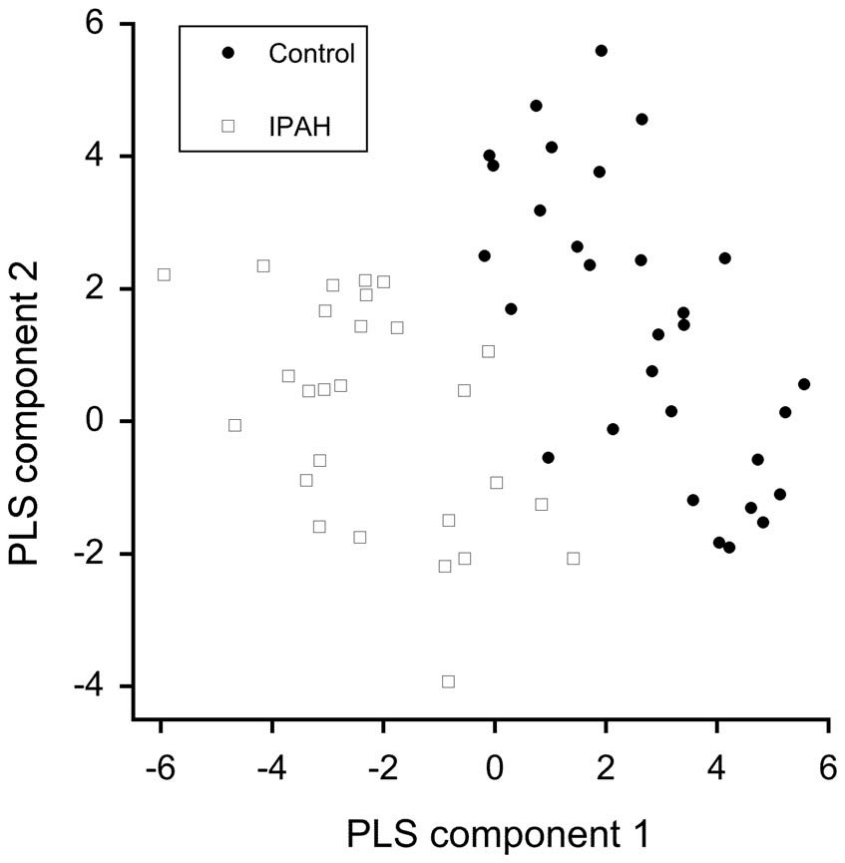
Plot of the results of autoregression and partial least squares analysis-weighted components of control subjects (dark circles) and IPAH subjects (open squares). 22 out of 27 disease (positive) samples were confirmed as positive (sensitivity  = 81.5%) and 21 out of 30 control (negative) samples were confirmed as negative (specificity  = 70.0%). The positive likelihood ratio was 2.76 while the negative likelihood ratio was 0.368.

The results of the GC/MS AR/PLSR analysis is shown in Figure 2. This analysis of the EBC metabolic signature shows that based on cross validation, the separation acurracy obtained by applying PLSR to AR coefficients is 75%. Using the pertinent information extracted from the whole spectra, the classification results suggest a good and robust seperability. Additionally, 22 out of 27 disease (positive) samples were confirmed as positive (sensitivity  = 81.5%) and 21 out of 30 control (negative) samples were confirmed as negative (specificity  = 70.0%). The positive likelihood ratio was 2.76 while the negative likelihood ratio was 0.368.

### Chemical Identification

Two peaks unique to the control group, 10 peaks unique to the IPAH group and 4 peaks common to both groups were identified chemically with high confidence (Table 3). An in-common peak at retention time of 75.479 min. had significantly greater ion counts (p = 0.011) in the control group compared to the IPAH group. This peak was identified as benzene, 1-methyl-4-(1-methylethnyl)-, a volatile organic compound produced by gut microbiota [19].

**Table 3:** Chemicals identified with “high confidence” for peaks found in all groups.

Peak Retention Time (min)	Present In	Proposed Chemical
**18.843**	IPAH	methyl isobutyl ketone
**29.003**	IPAH	furan, tetrahydro-2, 2, 4, 4-tetramethyl
**52.031**	Control	oxime-, methoxy-phenyl
**55.271**	In-common	benzaldehyde
**58.237**	IPAH	aniline
**58.730**	Contol	p-menth- 3 - ene
**60.262**	IPAH	2-menthene or other menthene isomers
**65.244**	IPAH	m-cymene or o-cymene
**71.801**	In-common	ethanone, 2,2-dihydroxy-1-phenyl-
**75.479**	In-common	benzene, 1-methyl-4-(1-methylethenyl)-
**81.436**	IPAH	benzenamine, N-ethyl-
**83.224**	IPAH	p-menthone
**96.547**	IPAH	benzothiazole
**124.314**	In-common	propanoic acid, 2-methyl-, 3-hydroxy-2, 4, 4-trimethylpentyl ester
**127.194**	IPAH	propanoic acid, 2-methyl-, 3-hydroxyhexyl ester
**157.789**	IPAH	1, 6-dioxacyclododecane-7, 12-dione

All peaks were identified with “high confidence”, i.e., these structures were more likely to be the correct match than other potential candidate compounds.

### Correlation and Regression Analysis

**Figure 3:**
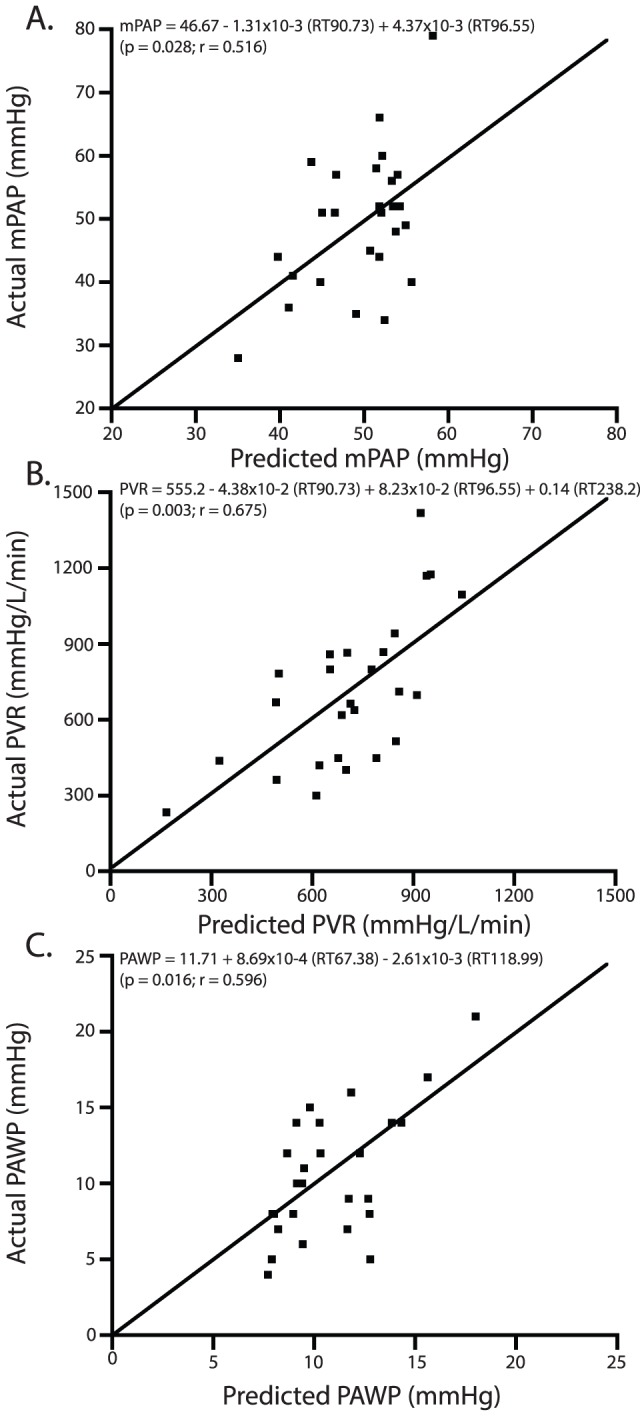
Plots of step-wise linear regression analysis of actual vs. predicted values for mPAP, PVR, and PAWP. There were significant associations between mPAP and peak heights at retention times of 90.733 and 96.547 minutes (A); PVR and peak heights at retention times of 90.733, 96.547 and 238.247 minutes (B); PAWP and peak heights at retention times of 67.380 and 118.995 minutes (C).

Six of the 32 IPAH unique peaks were correlated with mPAP, PVR or PAWP (Table 4). There were no significant correlations between BNP or ExNO and mPAP, PVR and PAWP (Table 4). mPAP, PVR, and PAWP were used in the step-wise linear regression analysis and are shown in Figure 3.

**Table 4:** Correlations with pulmonary hemodynamic variables.

RT (min) or Biomarker	mPAP	PVR	PAWP
**67.380**			0.429 (26; 0.029)
**81.436∧**	0.554 (14; 0.040)		
**90.733**	−0.423 (26; 0.031)	−0.476 (26; 0.014)	
**96.547∧**	0.475 (26; 0.014)	0.427 (26; 0.029)	
**118.995**			−0.497 (26; 0.019)
**238.247**		0.483 (26; 0.012)	
**ExNO**	−0.132 (25; 0.530)	−0.242 (25; 0.243)	−0.181 (25; 0.386)
**BNP**	−0.204 (23; 0.351)	−0.159 (23; 0.468)	0.273 (23; 0.208)

Pearson correlation coefficients r (number of subjects; p value); **∧** identified chemically with high confidence; RT = retention time; mPAP = mean pulmonary arterial pressure; PVR = pulmonary vascular resistance; PAWP = pulmonary arterial wedge pressure; ExNO = exhaled nitric oxide; BNP = brain naturetic peptide.

## Discussion

Currently, pulmonary hemodynamics along with 6MWD are common clinical measures used for the evaluation and diagnosis of IPAH [18]. In the current study the EBC metabolic signature was discriminatory in delineating severe IPAH subjects from age-matched controls. This in large part was due to the 48 peaks only present in IPAH subjects' gas chromatograms, and 62 peaks only present in control subjects' gas chromatograms. In addition, AR/PLSR analysis conserves these distinguishing features while greatly reducing the dimensionality of the data. This metabolomic approach provides a means to compare the complex information contained in the gas chromatograms and discriminate divergent groups, such as control and IPAH subjects in the current study. In total, these findings highlight the potential predictive value of VOCs in EBC for the evaluation and diagnosis of IPAH. This is supported by a positive likelihood ratio of 2.76 and a negative likelihood ratio of 0.37. Alternatively, the large number of unique compounds in both the control and IPAH groups suggests that an index for the screening of IPAH might be generated based on the presence or absence of a number of these compounds. However, the development and testing of such an index and the metabolomics approach based on AR/PLRS analysis requires further screening of a larger population of controls and individuals with diverse forms of pulmonary hypertension, as well as, individuals with other forms of lung disease to insure that the markers and chromatogram features identified are specific to IPAH.

The gas chromatograms obtained from IPAH samples would be expected to be affected by not only the underlying disease process but also the numerous medications the IPAH subjects were taking during the study. Of the 112 medications taken, 16 were taken by 4 or more of the IPAH subjects, indicating that most of the medications would not be expected to affect a large enough number of the 27 chromatograms studied to influence the group metabolic signature. The overall pattern is consistent with a metabolic signature that is unique to IPAH and is modified depending on the medications being taken. If any of the 48 IPAH unique peaks were a direct or indirect result of a medication or its metabolites, one would expect the height of those peaks to be different in subjects taking the medications compared with subjects not taking the medications. The data show that 30 of 48 IPAH unique peaks and 9 of 19 in-common peaks were affected by medication use; after eliminating peaks for individuals on given medications, 32 IPAH unique peaks and 14 in-common peaks contained a sufficient number of data points for subsequent correlation and regression analysis. Correlation and multiple linear regression analysis showed significant relationships between pulmonary hemodynamic PAH criteria and specific peaks from our GC/MS analysis (Table 4 and Figure 3). Although unknown at this point, it is possible that some medications affected peak heights in IPAH subjects and may have affected metabolic pathways important in the underlying mechanisms of IPAH.

Another factor that may have influenced our results is the significantly greater BMI in the IPAH group. This difference in BMI can be attributed to 4 subjects that had a mean BMI of 54.1. Removing these 4 subjects data from the dataset did not alter the outcomes of the AR/PLSR, correlation and regression analyses. This finding indicates that differences in BMI do not affect the unique characteristics of the chromatograms and individual peaks identified in our study groups using GC/MS.

The peak-by-peak analysis of GC/MS chromatograms provided insights into potential new biomarkers of disease severity and progression. Six peaks that were unique to the IPAH group significantly correlated with pulmonary hemodynamics (Table 4). Interestingly, many identified biomarker compounds exhibited definite structural similarities: a number of compounds differed by only one functional group or could be formed from one another by common cellular chemical reactions. Overall, the identified IPAH group specific compounds appear to be more oxidized than specific compounds identified in the control group. For example, several substituted arenes presented in Table 3 (benzenamine; N-ethyl-, aniline, oxime-; methoxy-phenyl, benzaldehyde; ethanone, 2,2-dihydroxy-1-phenyl-; benzene, 1-methyl-4-(1-methylethenyl)-) could easily be converted to each other or result from common precursors such as phenylalanine, tryptophan or tyrosine due to bacterial degradation. Of note, benzene, 1-methyl-4-(1-methylethenyl)- is found in both the control and IPAH groups and is significantly lower in the IPAH group. Also of particular interest is benzothiazole (RT = 96.547 min), that is unique to the IPAH group and is significantly correlated with mPAP and PVR (Table 4).

Recently, Cikach et al. [20] analyzed expired breath in patients with NYHA class I-IV PAH using selected ion flow tube-mass spectrometry. They identified low molecular mass compounds (ammonia, propanol and alkenes) that discriminated between their control and PAH groups and that correlated with clinical markers of disease. These authors carried out a quantitative assessment of 21 pre-specified VOCs that were selected based on being previously described in association to or in the context of PAH disease. These chemical species also needed to be suitable for analysis using SIFT MS. The common restrictions of the SIFT MS method are an upper limit of mass of usually 240 Th and the need for multiple reactive ions for tentative compound identification [21]. More importantly, the SIFT MS method does not allow for unambiguous identification of unknown species, especially those of higher molecular mass that can have greater number of isomers. In Cikach et al. [20], mass scanning of ion products for H3O+, O2+, and NO+ from 14 to 200 atomic mass units was performed. Thus, to accommodate molecular ions such as NO+M, the parent's mass could not be higher than approximately 170 Th. In the present study, the SPME sorbent (carboxen/polydimethylsiloxane) used for pre-concentration was optimized for molecular weight compounds of 30–225 Da. However, many compounds outside of this range can also be trapped. Additionally, the m/z range of the ion trap in the current study was set at 35–1000 Th ensuring inclusion of higher molecular weight biomarkers. By using this untargeted approach, the current study was able to identify several compounds not previously described and merit further investigation (Table 3). These structurally more complex molecules may convey more information regarding their source of origin and are less likely to result from background contamination [22]. Our subjects were NYHA class III and IV indicating that the metabolic profile of expired breath is altered in advanced IPAH. Additional studies that include NYHA functional class I and II patients would address whether the metabolites detected in the current study could be used in early detection of PAH.

The use of ExNO as a biomarker of IPAH has not always been successful partially due to variability seen in ExNO. ExNO has been reported to be decreased [23]–[25], normal [26], or increased [27] in PAH patients. In the current study, ExNO was significantly lower in IPAH patients compared to control subjects. ExNO was not correlated with any pulmonary hemodynamic endpoints in the current study. The present study included data on two previously described biomarkers of IPAH severity, plasma BNP and ExNO. Elevated plasma BNP or n-terminal proBNP has been shown to be correlated with the severity of right ventricular dysfunction in pulmonary hypertension [28]–[30]. In addition, BNP has been shown to be valuable as a prognostic indicator of IPAH [31] and is responsive to endothelin receptor antagonist treatment [32]. In contrast, our results show that BNP was not significantly correlated with any hemodynamic endpoints, including right atrial pressure. Given our restricted sampling of severe IPAH the lack of significant correlation with mPAP or any other hemodynamic measure should not be interpreted to mean that there is no relationship to BNP and the IPAH disease process. If a boarder range of diseases was present in our group or if BNP and hemodynamic values were available in our controls, then BNP may well have correlated with hemodynamic measures.

Our findings indicate the utility of EBC VOC analysis to discriminate between individuals with severe IPAH and age-matched healthy individuals and provides a means for identifying novel biomarkers significantly correlated with IPAH pulmonary hemodynamics. This novel metabolomics approach of analyzing VOCs in EBC in patients with IPAH using GC/MS provides a potential diagnostic tool for classification of disease severity and progression and will need to be confirmed in other subgroups of PAH that differ in severity and etiology to clarify its utility in disease management and detection.

## Supporting Information

Table S1 Concomitant medication/supplements list for IPAH subjects. Number of subjects taking each medication in parentheses.
(DOCX)
