# Supplementary material for: Analysis of Volatile Compounds in Exhaled Breath Condensate in Patients with Severe Pulmonary Arterial Hypertension
Source: PLoS One. 2014 Apr 18;9(4):e95331. doi: 10.1371/journal.pone.0095331 (PMC3991617; doi:10.1371/journal.pone.0095331)
Supplement: Table S1 — Concomitant medication/supplements list for IPAH subjects. Number of subjects taking each medication in parentheses. (DOCX) [file pone.0095331.s001.docx]

**Table S1. Concomitant medication list for IPAH Subjects. Number of subjects taking each medication in parentheses.**

Acetominophen (5)

Acyclovir (1)

Albuterol (5)

Allopurinol (2)

Alprazolam (1)

Ambrisentan (4)

Amiodarone (1)

Amitriptyline (2)

Amlodipine (3)

Amlodipine/benazepril (1)

Anusol cream (1)

Aspirin (6)

Atorvastatin (2)

Azithromycin (2)

B complex (2)

B12 (1)

Benazepril (2)

Bosentan (10)

Bupropion (2)

Calcium (1)

Carbidopa/Levodopa (1)

Carisoprodol (1)

Carvedilol (1)

Celecoxib (2)

Cinacalcet (1)

Clonazepam (1)

Colchicine (2)

Desvenlafaxine (1)

Dextropropoxyphene (1)

Digoxin (4)

Diltiazem (1)

Dipyridamole (5)

Docusate (2)

Duloxetine (1)

Enalapril (1)

Enoxaparin (1)

Esomeprazole (4)

Estradiol (1)

Ethacrynic acid (1)

Famotidine (2)

Febuxostat (1)

Felodipine (1)

Fenofibrate (1)

Fexofenadine (1)

Flax Seed Oil (1)

Fluoxetine (4)

Fluticasone (1)

Fluticasone/Salmeterol (2)

Fosinopril (1)

Furosemide (20)

Gabapentin (5)

Glibenclamide (1)

Glucosamine (1)

Guaifenesin (1)

Hydrocodone (1)

Hydrocodone/Acetaminophen (7)

Hydroxyzine (3)

Ibuprofen (3)

Iloprost (1)

Insulin (1)

Ipratropium (2)

Iron (4)

Iron sucrose (1)

Iron Supplement (1)

Lansoprazole (1)

Levalbuterol (1)

Levofloxacin (1)

Levothyroxine (6)

Lisinopril (2)

Lomotil (2)

Loperamide (1)

Lorazepam (1)

Lovastatin (1)

Magnesium (1)

Meclizine (1)

Metformin (1)

Metolazone (7)

Metoprolol (4)

Multi vitamin (3)

Omeprazole (3)

Ondansetron (4)

Oxycodone (2)

Oxygen (1)

Pantoprazole (2)

Potassium (5)

Pravastatin (2)

Prednisone (2)

Pregabalin (1)

Premarin (1)

Prochlorperazine (3)

Prostacyclin (1)

Rabeprazole (1)

Riociguat (1)

Sertraline (2)

Sildenafil (11)

Simvastatin (4)

Simvastatin/Niacin (1)

Spironolactone (19)

Steroid lotion (1)

Sulfacetamide (1)

Sumatriptan (2)

Tadalafil (2)

Tiotropium (1)

Tramadol (4)

Trazodone (2)

Treprostinil (20)

Triamcinolone (2)

Ubiquinone (1)

Vit B (2)

Vit C (1)

Wararin (17)

Zolpidem (1)
